# Case report: Left atrial myxoma with morphology of cavernous hemangioma supplied by the right coronary artery

**DOI:** 10.3389/fcvm.2023.1207339

**Published:** 2023-08-01

**Authors:** Shouji Zhang, Junlin Wang, Fahang Song, Fan Yang, Fang Li, Shangxin Liu, Jiwei Ma, Haizhou Zhang, Xiaochun Ma

**Affiliations:** ^1^ Department of Cardiovascular Surgery, Shandong Provincial Hospital Affiliated to Shandong First Medical University, Jinan, China; ^2^ Department of Cardiovascular Surgery, Shandong Provincial Hospital Affiliated to Shandong University, Jinan, China; ^3^ Department of Anesthesiology, Shandong Provincial Hospital Affiliated to Shandong First Medical University, Jinan, China; ^4^ Department of Anesthesiology, The First Affiliated Hospital of Shandong First Medical University & Shandong Provincial Qianfoshan Hospital, Shandong Institute of Anesthesia and Respiratory Critical Medicine, Jinan, China; ^5^ Imaging Department, Pingyin Chinese Medicine Hospital, Jinan, China; ^6^ Department of Pathology, Shandong Provincial Hospital Affiliated to Shandong First Medical University, Jinan, China

**Keywords:** cardiac tumors, cardiac myxoma, cardiac surgery, coronary steal, cardiac pathology, coronary angiography

## Abstract

Here, we report an unusual case of left atrial myxoma presented with morphology of cavernous hemangioma supplied by the right coronary artery. Surgical resection of the left atrium myxoma was performed, and the patient experienced an uneventful recovery during hospitalization.

## Introduction

The incidence of primary cardiac tumors has been reported to be as low as approximately 0.02% (1–5). Myxomas account for nearly 77% of all primary cardiac tumors (6–8). Echocardiography, cardiac MRI, coronary computed tomography angiography (CTA), and coronary angiography are available diagnostic modalities for cardiac myxoma (8–10). Here, we reported an uncommon case of an adult female patient in whom a left atrial myxoma presenting with partial morphology of cavernous hemangioma was supplied by the right coronary artery. Surgical treatment was performed to completely excise the myxoma. The patient had a smooth recovery following surgery and did not experience any significant complications during hospitalization.

## Case description

**Figure 1:**
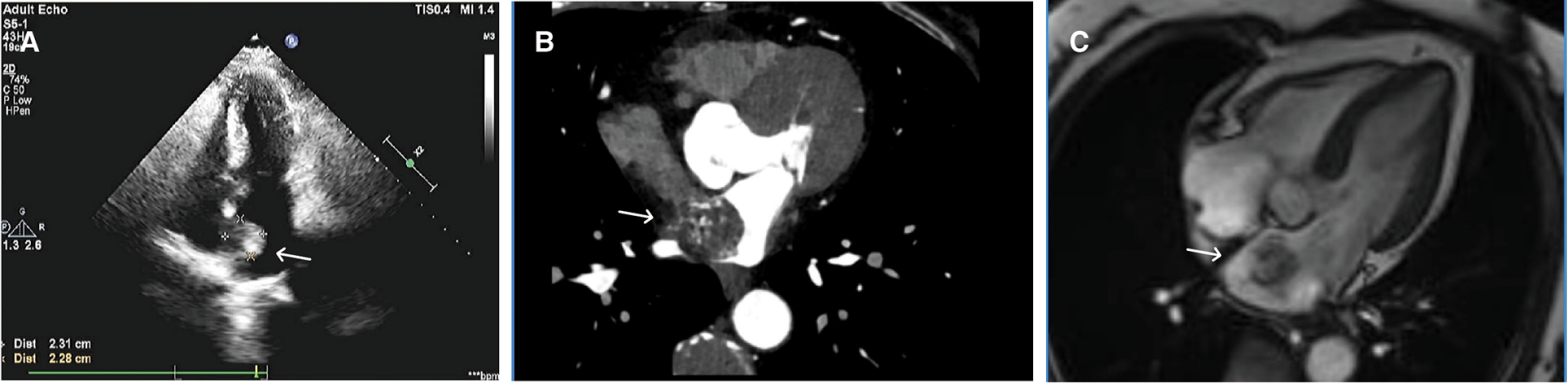
Preoperative echocardiography, coronary CTA, and cardiac MRI. (**A**) Echocardiography showed a medium echogenic mass attached to the upper part of the interatrial septum in the left atrium, with a wide base, regular shape, and fixed position. (**B**) Coronary CTA demonstrated a spherical lesion in the left atrium with right coronary artery blood supply. (**C**) LGE imaging depicted a spherical mass having a size of approximately 2.5 cm × 2.6 cm × 2.7 cm in the left atrium, with an abnormal signal showing a clear edge and patchy and uneven delayed enhancement.

**Figure 2:**
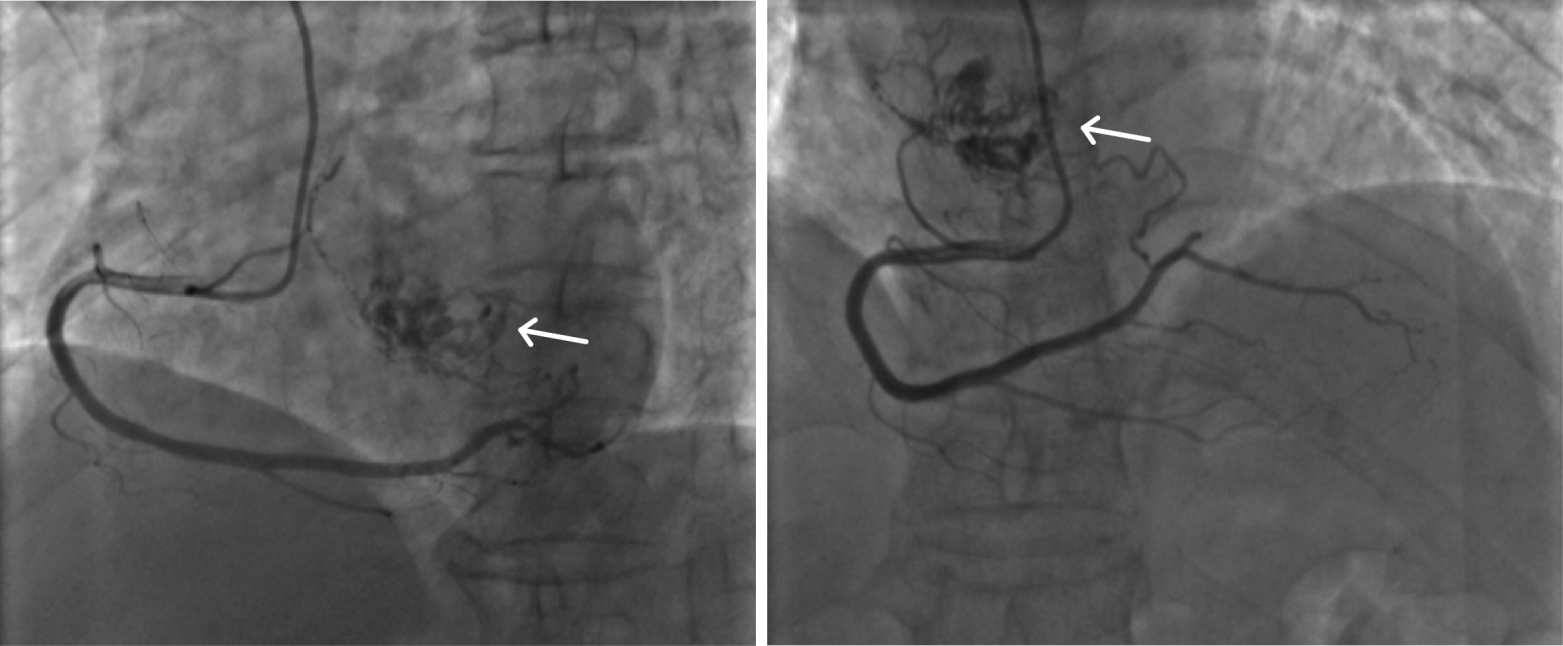
Characterization of the left atrial mass and its blood supply on coronary angiography. Preoperative coronary angiography detected a tumor-like mass in the left atrium and blood supply from the distal right coronary artery to the mass.

A 68-year-old female was admitted to the hospital with a chief complaint of paroxysmal chest tightness, shortness of breath, and palpitation for over a month. The patient had no history of hypertension or diabetes mellitus and other comorbidities. Preoperative echocardiography showed an intracardiac mass with medium-level echogenicity attached to the upper part of the interatrial septum in the left atrium, with a wide base, regular shape, and fixed position (Figure 1A). The coronary CTA showed a spherical lesion of equal density in the left atrium, closely related to the upper part of the interatrial septum, with right coronary artery blood supply (Figure 1B). Cardiac magnetic resonance imaging (MRI) depicted a spherical lesion with mixed isointense T1 and hyperintense T2 signal in the left atrium, with a clear edge and a size of approximately 2.5 cm × 2.6 cm × 2.7 cm. Late gadolinium-enhanced (LGE) imaging demonstrated patchy and uneven delayed enhancement in the left atrial mass (Figure 1C). Coronary angiography prior to surgery revealed the blood perfusion from the distal right coronary artery to the left atrial mass (Figure 2).

**Figure 3:**
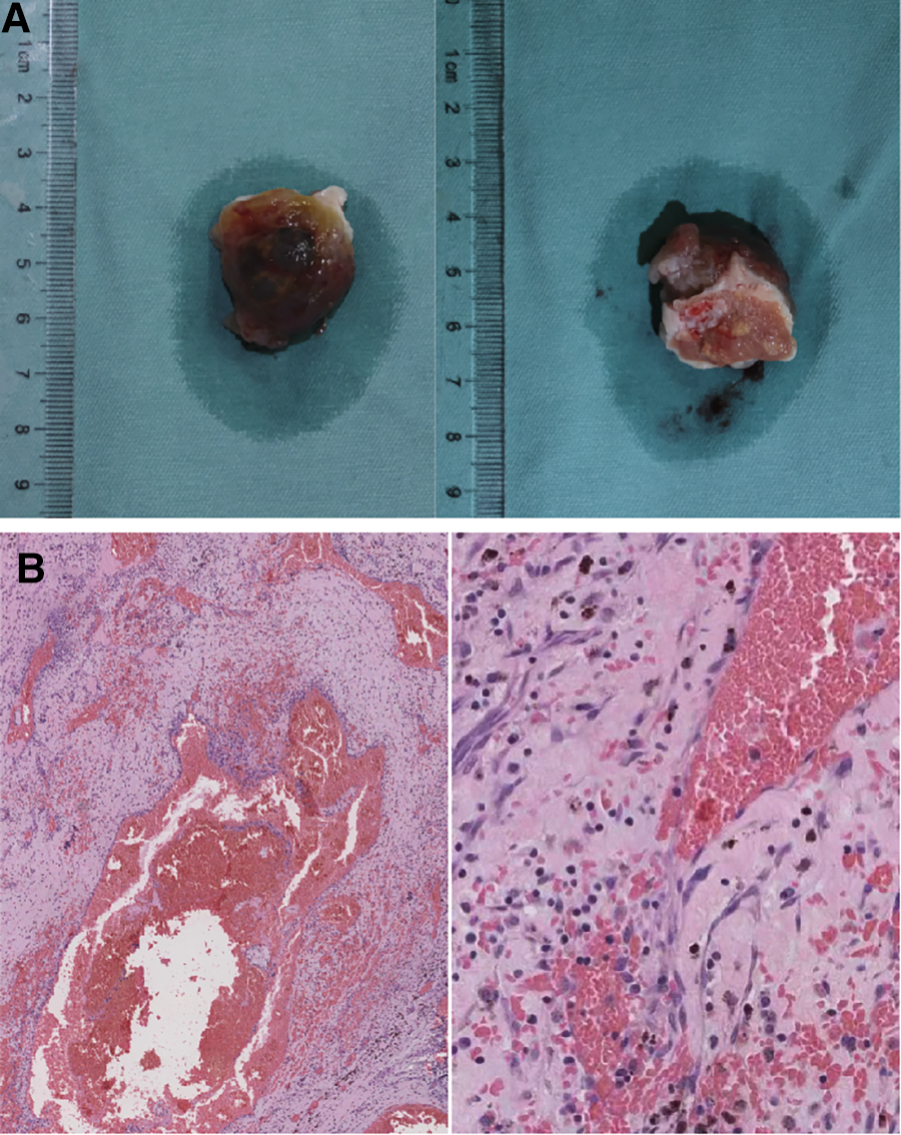
Gross pathological specimen and histopathology of the left atrial mass. (**A**) The mass resected from the left atrium. (**B**) Histopathology showed cavernous hemangioma with vascular dilation and congestion based on background of cardiac myxoma. The magnifications in (**B**) are ×40 (left) and ×200 (right).

The surgery was performed under general anesthesia with extracorporeal circulation. During the operation, the right atrium and interatrial septum were opened, revealing an irregular, nodular mass with a size of approximately 2.5 cm × 3.0 cm × 3.0 cm in the left atrium. The mass had a broad base and was located at the posterior and inferior portion of the interatrial septum, close to the orifice of the right inferior pulmonary vein. The mass also involved the posterior wall of the left atrium and was covered with a cyst-like, fluid-filled capsule. The lesion was completely excised along with a portion of the interatrial septum and posterior wall of the left atrium (Figure 3A). A bovine pericardium patch was used to repair the posterior wall of the left atrium and the atrial septal incision was continuously sutured. The surgery was successful, with aortic clamping and extracorporeal circulation lasting 40 and 61 min respectively.

**Figure 4:**
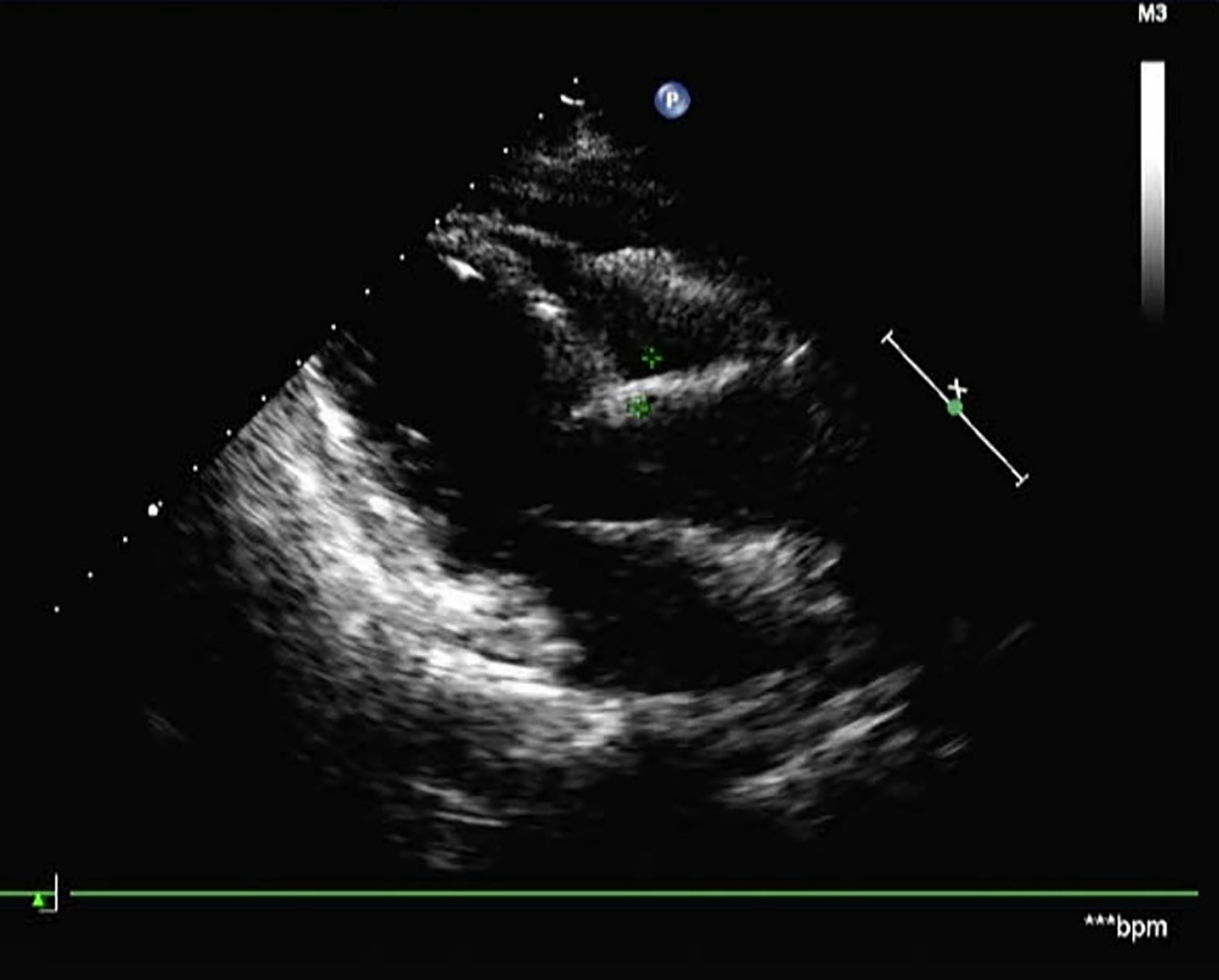
Postoperative echocardiography showed no residual tumor tissue and a normal cardiac chamber structure.

Postoperative histopathology demonstrated that the lesion tissue was a left atrial myxoma, presenting with partial cavernous hemangioma showing vascular dilation and congestion (Figure 3B). Postoperative echocardiography did not detect any residual tumor tissue, and cardiac chamber structure and function were normal (Figure 4).

The recovery following surgery was smooth, and the patient did not experience any significant complications during hospitalization. Upon discharge 6 days after surgery, the symptoms such as chest tightness and shortness of breath were completely relieved. During a 3-month follow-up, her chest tightness, shortness of breath, and palpitation had resolved completely. Echocardiography showed that the cardiac myxoma did not recur.

## Discussion

Cardiac myxoma is the most common type of adult primary heart tumor. In recent years, the possibility of misdiagnosis of cardiac myxoma has decreased significantly due to the utilization of diagnostic modality such as echocardiography, coronary CTA, and cardiac MRI (8–10). Coronary CTA and angiography could reveal the blood supply of cardiac myxoma (11, 12). Although cardiac myxoma is benign, it may grow toward the heart cavity and result in blood flow obstruction and heart valve dysfunction. The detachment of myxoma may even cause severe systemic embolism. Surgical incision of cardiac myxoma is required once the diagnosis is established, and cardiac myxoma may still recur after surgery; thus, regular postoperative follow-up is necessary.

Cardiac myxoma is also the most common benign tumor of the heart (13). Although cardiac cavernous hemangioma is a rare benign tumor, there have also been reports (14). However, the combination of cardiac myxoma and cavernous hemangioma has not been reported yet. In this case, the left atrial tumor included two portions: both myxoma and cavernous hemangioma. The myxoma could be observed in the area consisting of sparse irregular spindle-shaped and star-shaped fibroblasts, with small and deeply stained nuclei, scattered tumor cells, loose mucinous stroma, and varying numbers of small blood vessels. In the background of myxoma, thin-walled large blood vessels with different diameters could be detected. The wall of the vessels was composed of flat endothelial cells without atypia, with smooth muscles also visible. Thrombosis could be noticed in the vascular lumen, and fibrous septa seen in the large lumen. This case needs to be differentiated from simple atrial myxoma, which also shows blood vessels, but mostly thick-walled small vessels and rarely thin-walled large vessels (15, 16). Cavernous hemangioma also needs to be distinguished from well-differentiated angiosarcoma. Angiosarcoma often manifests infiltrative growth, with vascular endothelial cells arranged in an atypical or multiple layers and visible nuclear mitotic, and has a poor prognosis (17).

Both myxomas and hemangiomas belong to mesenchymal tumors and thus may have the same origin. The ultrastructural examination of cardiac myxoma shows that it is derived from multipotential mesenchymal cells. On the one hand, immunohistochemistry indicates that myxoma expresses vascular endothelial markers, such as CD31 and CD34, which are considered markers of mesenchymal cells differentiating into endothelial cells (18). On the other hand, studies have found that myxoma expresses epithelial markers such as carcinoembryonic antigen (CEA) and epithelial membrane antibody (EMA), and cardiomyocyte-specific transcription factors, which indicate myocardial differentiation (19). Overall, myxoid of cardiac myxoma may originate from mesenchymal cells with multi-directional differentiation potential. These mesenchymal cells can not only differentiate into myxoid cells but also secrete angiogenic factors such as vascular endothelial growth factor (VEGF), promoting angiogenesis. Finally, the myxoma has a rich angioma-like thin-walled vessel filled with mucus such as chondroitin sulfate at the same time, forming a morphological myxoma structure (20). Hemangiomas arise from multipotent stem cells and these hemangioma stem cells that give rise to the endothelial cells are also the essential source of adipocytes during hemangioma involution (21). Based on the two types of morphologies, myxomas and hemangiomas are still considered independent diseases and we state that our case has its special pathological features.

Coronary angiography is a common and effective method for diagnosing coronary artery disease and is a safe and reliable invasive diagnostic technique, which is now widely used in clinical practice. Preoperative coronary angiography for cardiac myxoma patients not only excludes coronary artery disease but also detects the artery feeding the cardiac myxoma. In a review of coronary angiography in 19 patients with cardiac myxoma, seven had angiographically visible tumor vascularity. One patient had significant coronary artery disease of the circumflex coronary artery and another patient had a thrombus-like lesion in the proximal third of the left anterior descending coronary artery (22). In another retrospective study of 42 cardiac myxoma patients performed by Lee et al., 21 (50%) of the patients were discovered to have artery feeding to the myxoma. The feeding arteries in this study included right coronary artery (RCA) (57.1%), left circumflex coronary artery (LCx) (28.6%), and both (14.3%) (23). In addition, coronary angiography can reveal the distinctive morphology of the myxoma feeding artery, which can be helpful in surgical planning (24). Indeed cardiac myxomas having blood supply from the right coronary artery are not uncommon, since the blood perfusion of atrial septum depends upon the dominant right coronary artery pattern. However, we state that in this case, the myxoma has a unique feature of rich and extensive blood supply from the right coronary artery. First, coronary angiography showed a more dense distribution of vessels in the myxoma. LGE imaging also demonstrated obvious, patchy, and uneven delayed enhancement in the left atrial mass. In addition, sufficient blood supply of the myxoma leads to the coronary steal which may explain the paroxysmal chest tightness and palpitation symptoms of the patient.

Stiver et al. reported a case of a large intracardiac left atrial mass supplied by two anomalous coronary arteries causing coronary steal (25). Coronary steal, a rare phenomenon of unbalanced blood flow, is primarily seen in patients with prior coronary artery bypass grafting specifically in the left internal mammary artery (LIMA) to the left anterior descending artery (LAD) graft and simultaneously with significant subclavian artery stenosis. Very few cases reported that cardiac myxomas supplied by coronary arteries can cause coronary steal, even though no coronary lesions were observed in coronary angiography (25–27). First, patients may present with fatigue, weakness, exertional chest heaviness, or angina, and coronary steal explains the symptoms of cardiac ischemia. Second, it was reported that the mechanisms of coronary steal included highly vascularized myxoma (neovascularization), hemorrhage, and associated fistula formation in myxoma. Coronary angiography reveals the details of the blood supply. It is remarkable and verifiable that not only the total incision of the myxoma but also the ligation of the supplying branches helps solving the symptoms of patients (25–27).

## Data Availability

The original contributions presented in the study are included in the article/Supplementary Material, further inquiries can be directed to the corresponding authors.
